# Clinical study of diabetic peripheral neuropathy screening by retinal vascular geometric parameters

**DOI:** 10.1038/s41598-021-85831-0

**Published:** 2021-03-24

**Authors:** Guotao Hu, Hongmei Wu, Lei Kuang, Benny Chung-Ying Zee, Ying Huang, Zhen Huang, Li He, Yuanhong Zeng, Yongbo Gao, Hailan Wang

**Affiliations:** 1 Department of Endocrinology, Longgang Central Hospital, Shenzhen 1228 Longgang Road, Shenzhen, 518116 Guangdong China; 2 Department of Biostatistics, The Jockey Club School of Public Health and Primary Care, The Chinese University of Hong Kong, Shatin, New Territories Hong Kong, China; 3 Department of Laboratory, Longgang Central Hospital, Shenzhen, 518116 Guangdong China; 4 Department of Nursing, Longgang Central Hospital, Shenzhen, 518116 Guangdong China; 5 Department of Ophthalmology, Longgang Central Hospital, Shenzhen, 518116 Guangdong China; 6 Department of Stomatology, Longgang Central Hospital, Shenzhen, 518116 Guangdong China

**Keywords:** Diabetes, Eye diseases

## Abstract

To investigate the relationship between geometrical changes of retinal vessels and diabetic peripheral neuropathy **(**DPN), and to determine the effectiveness of retinal vascular geometry analysis and vibration perception threshold (VPT) for DPN assessment. Type 2 diabetes patients (n = 242) were categorized by stage of DPN. VPT and fundus photography was performed to obtain retinal vascular geometry parameters. The risk factors for DPN and the correlation between DPN stages were analyzed. The efficiency of the retinal vascular geometric parameters obtained with VPT as a diagnostic tool for DPN was examined. Stages of DPN showed a linear correlation with VPT (r = 0.818), central retinal vein equivalent (CRVE) (r = 0.716), and fractal dimension arterioles (DFa) (r = − 0.769). VPT, CRVE, DFa, and fractal dimension veins (DFv) showed high sensitivity (80%, 55%, 82%, and 67%, respectively) and specificity (92%, 93%, 82%, and 80%, respectively) for DPN diagnosis. Good agreement was observed between combined use of geometric parameters (CRVE, DFa and DFv) and VPT (Kappa value 0.430). The detection rate of DPN with combined use of geometric parameters of retinal vessels (64.88%) was significantly higher than that with use of VPT (47.52%). Retinal vascular geometry changes demonstrated significant correlation with DPN severity. VPT, CRVE, DFa, and DFv may provide insights for understanding DPN.

## Introduction

Diabetic peripheral neuropathy (DPN) is a common complication of diabetes with various manifestations. DPN shows few clinical symptoms or manifestations in early stages. The severity of DPN is often not reflected by the manifestations. Considerable discrepancy has been observed between the incidence of DPN reported from clinical studies and population-based studies^1–4^. A large proportion of patients are not diagnosed until irreversible demyelination, pathological changes in peripheral nerves, or other clinical symptoms occur^5^. Therefore, early screening, diagnosis and intervention for DPN is crucial for the control of neuropathological changes^6^.

Early diabetes is often associated with small myelin lesions. While nerve conduction velocity (NCV) is considered to be the gold standard for the diagnosis of DPN^7^, NCV does not detect abnormality of small unmyelinated fibres. Other scoring systems, including *Toronto clinical scoring system (TCSS)*^8^, *Michigan neuropathy screening instrument (MNSI)*^9^, *Neurologic Deficit Score (NDS)*^10^, *Diabetic Neuropathy Symptom Score (DNSS)*^11^ are simple, but tend to be heavily influenced by subjective factors and have poor reproducibility. Studies have shown that these scoring systems are of limited diagnostic value for screening asymptomatic neuropathy^12^. In contrast, vibration perception threshold (VPT) is an effective DPN screening method with the ability to evaluate the risk of foot ulcers^13^. VPT detects and evaluates the vibration sensation in functional myelinated nerve fibers. It has been widely used clinically for diabetic neuropathy screening in United States and Europe. American diabetes association (ADA) recommends the use of VPT for diagnosis of DPN^14^.

Microvascular disease is the basis of a variety of complications in diabetes. In our previous investigation, we found changes in microvascular permeability and the expression of vascular endothelial growth factor in the sciatic nerve of diabetic rats. Our findings demonstrated the role of microvascular permeability in the pathogenesis of diabetic peripheral neuropathy^15^. A close association between microcirculation dysfunction and DPN was also demonstrated^15^, which is in line with other studies^16,17^. Thus, microvascular disease and peripheral nerve dysfunction are interrelated. Retinal microvascular and cardiovascular system have common anatomical characteristics. However, unlike cardiovascular system, retinal microvasculature can be directly examined in a non-invasive manner. Fundus photography, which is the most commonly used method for retinal microvascular imaging, reveals normal, altered and pathological vasculature.

Retinal vascular alteration is closely correlated to DPN. Ding et al*.*^18^ reported the correlation between retinal microvascular abnormalities and DPN with computer assisted program retinal vascular caliber (CRAE, CRVE) on fundus photography. The association between large arterial branches coefficients and DPN has also been reported. In a recent study by Rasmussen et al*.*^19^, branching coefficient veins were shown to be one of the risk factors for DPN. These early studies demonstrated an association between changes in retinal blood vessels and DPN. However, the effectiveness of the examination of retinal vessel geometry for screening of DPN has not been considered.

We hypothesized that retinal vessel geometry parameters would be useful for the diagnosis and screening of DPN. In this clinical study, patients with diabetes were enrolled, scored and grouped according to *modified Dyck staging*^20^. General clinical information, VPT and different retinal vessel geometry parameters were acquired. Different clinical measurements were compared and correlated in different stages of DPN to investigate the use of retinal vessel geometry parameters for screening and diagnosis of DPN and to provide reference measurements for clinical research.

## Subjects and methods

### Materials

The main instruments included Mydriatic TRC NW 300 fundus camera (Topcon Co. Ltd., Japan), Bio-Thesiometer quantitative sensiometer (China Beijing Di Meide Technology Co. Ltd., China), and screening diagnostic kit for diabetes complications (Japan Lin Electric Co. Ltd., Japan).

### Patients

Patients with type-II diabetes mellitus who were diagnosed based on the WHO criteria^21^, and who were admitted between February 2015 and September 2016 at the Department of Endocrinology of Shenzhen Longgang Hospital were included in this study. A total of 242 patients (162 men and 80 women; mean age: 48.80 ± 11.29 years) were selected by screening criteria. The mean duration of diabetes mellitus was 5.69 ± 5.57 years and the mean glycosylated hemoglobin level was 11.02 ± 2.71%. A total of 132 patients (83 men, 49 women) were found to have DPN. Informed consent forms were signed by patients and the health controls. This study was approved by the Shenzhen Longgang Hospital Ethics Committee and all methods were performed in accordance with the relevant guidelines and regulations.

### Exclusion criteria

Patients with gestational diabetes and diabetes associated with iatrogenic Cushing's syndrome, Sheehan syndrome, or adrenal syndrome abnormalities were not included. Patients with history of refractory hypertension, secondary hypertension, chronic liver and kidney disease, infectious diseases, malnutrition, connective tissue disease, mental illness, thyroid disease, and other endocrine diseases were also excluded. Patients aged over 80 years were excluded to avoid the influence of age-related complications.

Cases of pernicious anemia, vitamin B6 poisoning, alcoholism, uremia, chemical toxins, nerve compression and, hepatitis, idiopathic neuropathy, congenital disease (hereditary sensation, motor neuropathy), paraneoplastic syndrome, syphilis, HIV/AIDS, drugs (e.g. chemotherapy drugs, isoniazid), cervical lumbar spine (nerve root compression, spinal stenosis, cervical and lumbar degeneration), cerebral infarction, Guillain–Barre syndrome, and severe arteriovenous vascular disease (venous thrombosis, lymphangitis, arteriovenous fistula) were also excluded.

Patients with eye diseases such as acute and chronic corneal disease, glaucoma, traumatic eye disease, uveitis, vitreous hemorrhage and moderate to severe cataract were excluded. Poor fundus image quality, which hindered the analysis of the characteristics of blood vessels were rejected. Patients with diabetic retinopathy were not excluded; however, patients who had undergone prior panretinal photocoagulation (PRP) were excluded.

The control group in this study consisted of 39 healthy individuals with no history of any of the following: diabetes mellitus; hypertension; mental disorder; diseases of the nervous, hematologic, cardiovascular or cerebrovascular systems; autoimmune diseases. All subjects in the control group underwent routine blood, urine, and stool examination and were found to have normal liver and kidney function as well as blood lipids. No abnormalities were detected on physical examination, chest radiography, electrocardiogram, abdominal ultrasonography, and fundus examination.

### Research methods

#### General information and related biochemical tests

General information including age, age, sex, duration of diabetes, height, weight, blood pressure, body mass index (BMI) and history of smoking and drinking were recorded.

Fasting blood glucose, glycosylated hemoglobin (HbAIc), fasting C-peptide, total cholesterol, triglycerides, HDL, LDL, serum creatinine, uric acid, urea nitrogen were examined. Urine was collected in the morning every day for the assessment of 24-h urinary protein (UAE) and excretion rate (UAER).

#### Diagnosis of diabetic neuropathy and patient grouping

Diagnostic criteria: Definite history of diabetes or evidence of abnormal glucose metabolism with neuropathy during or after the diagnosis of diabetes mellitus. *Michigan Neuropathy Screening Instrument* *(MNSI)* was used for patient symptom scoring. Neurological deficits score (NDS) was used for patients signs scoring. Patients with MNSI symptom score ≥ 4; or MNSI sign score ≥ 2 with NDS ≥ 6; or NDS ≥ 3 with MNSI symptom score ≥ 4, were diagnosed with DPN.

According to the *modified Dyck staging*^20^, patients were divided into 4 groups. Stage 0 DPN (no evidence of diabetic neuropathy); Stage 1 DPN (no symptoms with abnormal signs); Stage 2 DPN (symptomatic with signs of ankle dorsiflexion weakness); Stage 3 DPN (disabling neuropathy, diabetic foot, etc.).

#### Vibration perception threshold measurement

Bio-Thesiometer quantitative sensiometer *(China, Beijing IndyMac Seoul Technology Co., Ltd.)* was used with reference to the international working group on the diabetic foot (IWGDF)^22^. Patients were advised to familiarize themselves with the vibratory sensation and to inform examiner for the vibratory sensation without much adaptation. The rubber tactor was balanced vertically on the pulp of the big toe. The voltage was increased from 0 V till the vibratory sensation. The test was repeated 3 times on each foot. The average VPT of both feet was recorded.

### Fundus photography and retinal vessel analysis

**Figure 1:**
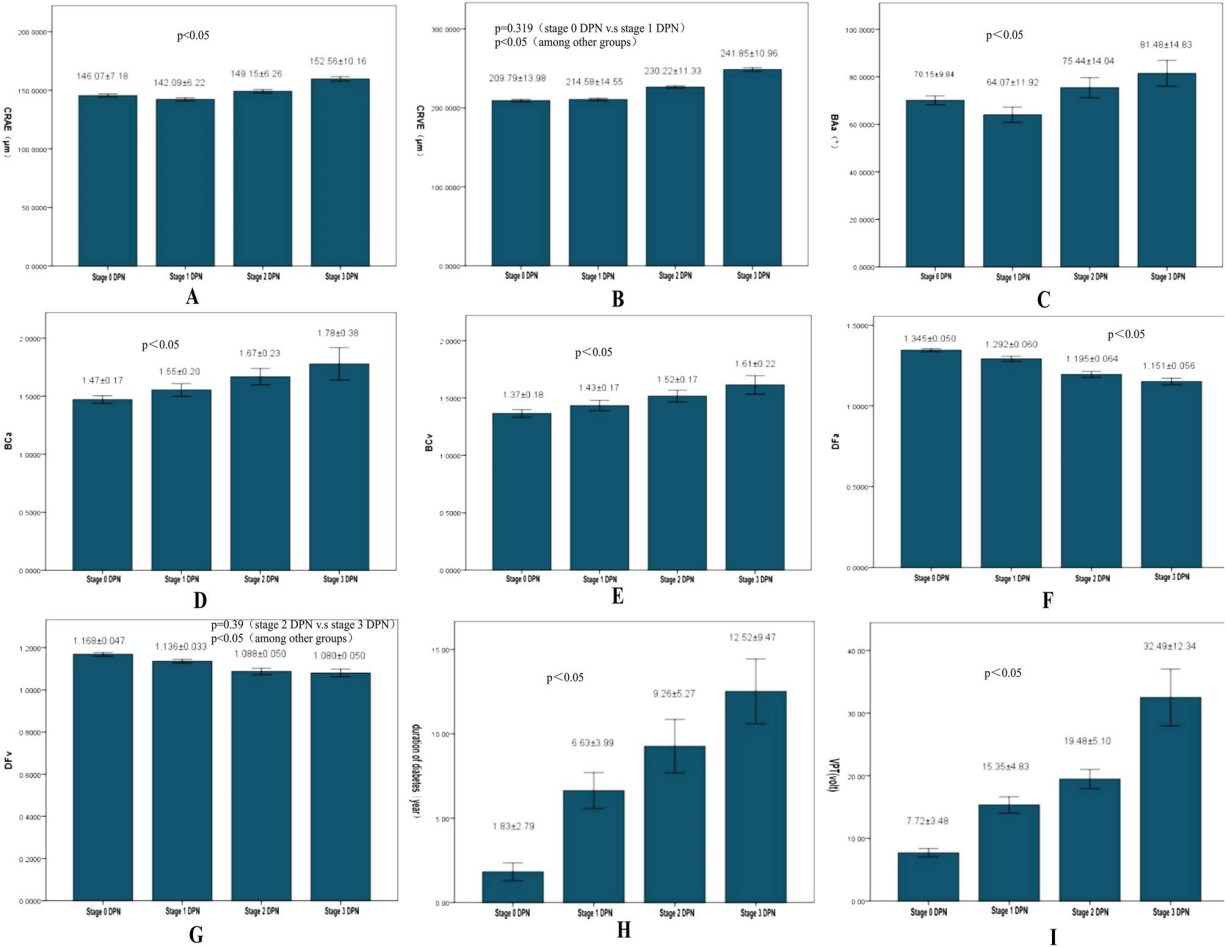
CRAE, CRVE, BAa, BCa, BCv, DFa, DFv, duration of diabetes, and VPT in different DPN stages. *CRAE* Central retinal artery equivalent, *CRVE* Central retinal vein equivalent, *Baa* Branching angle arterioles, *BCa* Branching coefficient arterioles, *BAv* Branching angle veins, *BCv* Branching coefficient veins, *DFa* Fractal dimension arterioles, *DFv* Fractal dimension veins, *VPT* Vibration perception threshold, *DPN* diabetic peripheral neuropathy.

Pupils were dilated with 0.1% Guttae Atropini Sulfatis before ophthalmic examination performed by an ophthalmologist. TRC NW 300 mydriatic fundus camera *(Japan TOPCON Corporation)* was employed to acquire color fundus photography at 45°. Disc centred (or optic nerve head centred) retinal photographs were obtained at maximum 1.5 times of disk diameter (Fig. 1a–c). The magnification of photograph was approximately ×15^23^. At least one fundus photograph of each eye was obtained and saved as TIFF 1365 × 1024 pixel format for retinal vessel analysis. Since the left and right ocular vessel geometric measurements show a strong correlation^24^, the fundus photographs from the right eye were analyzed. Thirty five fundus photographs from the right eye were replaced with left eye due to image quality issue, or due to vascular recognition software error.

The circumference of optic disc was used to adjust magnification efficiency (image conversion factor, ICF value). ICF value was extracted from Image J (version 1.52; URL: https://imagej.en.softonic.com/) and imported into Singapore retinal image analysis system (Singapore "I" Vessel Assessment, SIVA) in collaboration with the Chinese University of Hong Kong, for the analysis of retinal vessel parameters. The retinal vessel parameters were as follows (Supplementary Table S1),

1. Central Retinal Artery Equivalent (CRAE): The diameter of 6 broadest arterioles found in the range of 0.5–2 optic disc diameter. CRAE reflects changes in caliber of retinal arteries.
2. Central Retinal Vein Equivalent (CRVE): The diameter of 6 broadest veins found in the range of 0.5–2 optic disc diameter. CRVE reflects changes in caliber of retinal veins.
3. Branching Angle Arterioles (BAa): The angle of the upper two sub arteries bifurcation. Branch angle (normal value of about 75°) is associated with the number of vascular branches and the degree of tortuousity. Changes in BAa suggest hemodynamic changes, endothelial dysfunction, or altered blood oxygen concentration.
4. Branching Coefficient Arterioles (BCa): The ratio between the square of diameter of the central artery and the square diameter of the superior artery. Increased branching coefficient represents increased branch blood vessels or superior vascularization.
5. Branching Angle Veins (BAv): The angle of the upper two sub veins bifurcation.
6. Branching Coefficient Veins (BCv): The ratio between the square of diameter of the central vein and the square diameter of the superior vein.
7. Fractal Dimension Arterioles (DFa): The degree of filling of the retinal arterioles in the retinal plane. DF reflects the complexity of vascular and vessel density; the greater the value the more complex the structure.
8. Fractal Dimension Veins (DFv): The degree of filling of the retinal veins in the retinal plane.

### Statistical analysis

All statistical analysis was performed using SPSS software (version no. 22.0, URL: http://office.rjxzba.cn/goods.php?id=38&show=install). Quantitative data are presented as mean ± standard deviation (SD). Qualitative data are expressed as percentage. Independent sample *t*-test was applied for quantitative comparison between two groups, whereas One-way ANOVA was used for multiple group comparisons. Least significant difference (LSD) was used for pairwise multiple comparisons. Chi-squared test (χ^2^) was applied for qualitative analysis.

Pearson correlation analysis was employed for quantitative data, whereas Spearman correlation analysis was used for qualitative data. Logistic regression analysis was applied for multivariate regression analysis. SPSS software was used to assess the normality of distribution of each variable or to approximately subordinate to a near-field distribution. For all non-normally distributed variables, log-transformation was applied in SPSS software.

Receiver operating characteristic (ROC) curve analysis was performed and area under the curve (AUC) was determined. *Kappa* value was used for the assessment of DPN diagnostic agreement. P < 0.05 was considered as statistically significant.

### Ethics approval and consent to participate

This study was approved by the Shenzhen Longgang Hospital Ethics Committee and all methods were performed in accordance with the relevant guidelines and regulations. Informed consent forms were signed by patients and the health controls.

## Results

### General clinical information of diabetic patients

A total of 242 diabetic patients (162 men; 66.9%) with average age of 48.80 ± 11.29 years were included. Mean duration of diabetes mellitus was 5.69 ± 5.57 years and mean glycosylated hemoglobin was 11.02 ± 2.71%. A total of 39 healthy individuals (24 men and 15 women; mean age: 35.50 ± 7.21 years) were enrolled as the control group.

As shown in Table 1, 132 diabetic patients were diagnosed as DPN (incidence rate: 54.5%). Of these 83 (62.88%) were men. Stage 0 to 3 DPN was found to be 110, 56, 45 and 31, respectively. Male patients accounted for at least half of the population among the 4 stages. Age, duration of diabetes, fasting C-peptide, blood urea nitrogen, 24-h urinary protein, 24-h urinary protein excretion rate, vibration perception threshold and retinal vessel geometric parameters, including CRAE, CRVE, DFa, DFv, BCa, BCV, BAa, BAv, all showed significant difference in ANOVA comparison.

**Table 1:** General characteristics of diabetic patients disaggregated by stage of DPN.

Parameter	Control group	Stage 0 DPN	Stage 1 DPN	Stage 2 DPN	Stage 3 DPN	*P* value
Number (n = 242)	39	110	56	45	31	–
Male	24 (61.5%)	79 (71.8%)	40 (71.4%)	25 (55.6%)	18 (58.1%)	–
Age (years)	35.50 ± 7.21	44.05 ± 10.63	48.50 ± 10.15	54.53 ± 9.02	57.84 ± 9.47	** < 0.001 **^**※**^
Duration of diabetes (years)	0	1.83 ± 2.79	6.63 ± 3.99	9.26 ± 5.27	12.52 ± 5.23	** < 0.001 **^**※**^
Smoking	0	32 (29.1%)	10 (17.9%)	4 (8.9%)	4 (12.9%)	–
Drinking	0	47 (42.7%)	19 (33.9%)	10 (22.2%)	7 (22.6%)	–
BMI (kg/m^2^)	23.38 ± 0.65	24.30 ± 3.64	23.19 ± 3.67	23.29 ± 3.15	23.64 ± 2.52	0.164
Systolic blood pressure (mmHg)	118 ± 12.05	128.32 ± 18.88	133.64 ± 23.59	131.31 ± 3.18	138.97 ± 25.88	0.084
Diastolic blood pressure (mmHg)	75.88 ± 14.12	80.20 ± 12.11	80.25 ± 14.56	77.80 ± 13.97	76.84 ± 12.04	0.474
Glucose (mmol/L)	4.50 ± 1.45	11.78 ± 12.84	10.25 ± 3.39	11.14 ± 4.20	10.91 ± 2.80	0.752
HbA1c (%)	4.61 ± 1.39	11.20 ± 2.77	10.64 ± 2.38	11.11 ± 3.43	10.98 ± 1.82	0.559
Fasting C-peptide (ng/mL)	5.36 ± 1.14	2.36 ± 1.41	1.77 ± 1.13	1.94 ± 1.40	1.92 ± 0.82	**0.046 **^**※**^
TC (mmol/L)	3.55 ± 1.42	4.98 ± 1.72	4.93 ± 1.60	4.91 ± 1.71	5.37 ± 1.73	0.645
TG (mmol/L)	1.01 ± 0.84	2.80 ± 5.84	1.86 ± 2.85	2.33 ± 2.87	2.09 ± 2.83	0.585
HDL-C (mmol/L)	2.05 ± 0.48	1.10 ± 0.47	1.16 ± 0.40	1.08 ± 0.46	1.278 ± 0.32	0.072
LDL-C (mmol/L)	2.13 ± 0.77	4.42 ± 13.18	3.18 ± 1.26	3.19 ± 1.07	3.51 ± 1.16	0.791
Creatinine (μmol/L)	67 ± 15.07	81.16 ± 59.50	71.37 ± 25.47	81.73 ± 46.10	88.32 ± 33.84	0.424
Uric acid (μmol/L)	280 ± 48.21	339.08 ± 118.21	288.46 ± 91.96	332.23 ± 135.82	337.62 ± 103.52	0.061
BUN (mmol/L)	4.55 ± 2.08	5.71 ± 4.80	5.88 ± 6.26	5.57 ± 2.95	15.98 ± 50.93	**0.049 **^**※**^
UAE (mg)	11.45 ± 4.48	20.66 ± 54.98	57.15 ± 100.65	125.59 ± 356.36	352.47 ± 621.93	** < 0.001 **^**※**^
UAER (ng/L)	9.80 ± 6.87	15.08 ± 38.07	41.33 ± 70.11	91.54 ± 253.67	257.25 ± 429.30	** < 0.001 **^**※**^
VPT (V)	3.15 ± 3.34	7.72 ± 3.48	15.35 ± 4.83	19.48 ± 5.10	32.49 ± 12.34	** < 0.001 **^**※**^
ABI	1.102 ± 0.084	1.183 ± 0.036	1.112 ± 0.048	1.117 ± 0.074	1.109 ± 0.082	** < 0.001 **^**※**^
CRAE (μm)	147.02 ± 5.08	146.07 ± 7.18	142.09 ± 6.22	149.15 ± 6.26	152.56 ± 10.16	** < 0.001 **^**※**^
CRVE (μm)	198.78 ± 10.02	209.79 ± 13.98	214.58 ± 14.55	230.22 ± 11.33	241.85 ± 10.96	** < 0.001 **^**※**^
DFa	1.442 ± 0.050	1.345 ± 0.050	1.292 ± 0.060	1.195 ± 0.064	1.151 ± 0.056	** < 0.001 **^**※**^
DFv	1.183 ± 0.027	1.168 ± 0.047	1.136 ± 0.033	1.088 ± 0.050	1.080 ± 0.050	** < 0.001 **^**※**^
BCa	1.35 ± 0.15	1.47 ± 0.17	1.55 ± 0.20	1.67 ± 0.23	1.78 ± 0.38	** < 0.001 **^**※**^
BCv	1.28 ± 0.12	1.37 ± 0.18	1.43 ± 0.17	1.52 ± 0.17	1.61 ± 0.22	** < 0.001 **^**※**^
BAa (°)	71.16 ± 8.84	70.15 ± 9.84	64.07 ± 11.92	75.44 ± 14.04	81.48 ± 14.83	** < 0.001 **^**※**^
BAv (°)	80.12 ± 4.28	77.72 ± 14.71	74.29 ± 12.59	71.75 ± 14.14	68.39 ± 14.92	**0.005 **^**※**^

^※^Indicates one-way ANOVA, *P* < 0.05, statistically significant.

*DPN* diabetic peripheral neuropathy, *BMI* body mass index, *HbA1c* glycosylated hemoglobin, *TC* total cholesterol, *TG* triglycerides, *LDL-C* low-density lipoprotein cholesterol, *HDL-C* high-density lipoprotein cholesterol, *BUN* blood urea nitrogen, *UAE* 24-h urinary protein quantity, *UAER* 24-h urinary protein excretion rate, *VPT* Vibration perception threshold., *ABI* Ankle brachial index, *CRAE* Central retinal artery equivalent, *CRVE* Central retinal vein equivalent, *BAa* Branching angle arterioles, *BCa* Branching coefficient arterioles, *BAv* Branching angle veins, *BCv* Branching coefficient veins, *DFa* Fractal dimension arterioles, *DFv* Fractal dimension veins.

### Retinal vessel geometric parameters in different stages of DPN

CRAE was found to be significantly different between different stages of DPN and decreased in stage 1 and increased with the severity of DPN as shown in Fig. 1A. CRVE was found to be significantly different between different stages of DPN and increased with the severity of DPN, as shown in Fig. 1B. BAa was found to be significantly different between different stages of DPN and increased with the severity of DPN, as shown in Fig. 1C. BAv tended to decrease with severity of DPN. In particular, BAv in stage 2 and 3 DPN was significantly smaller than that in stage 0 DPN. BCa and BCv were found to be significantly different between different stages of DPN and increased with increase in the severity of DPN, as shown in Fig. 1D,E, respectively. DFa was found to be significantly different between different stages of DPN and decreased with increase in the severity of DPN, as shown in Fig. 1F. DFv was found to be significantly different between different stages of DPN, with the exception of stages 2 and 3, and decreased with increase in the severity of DPN, as shown in Fig. 1G. Duration of diabetes and VPT were found to be significantly different between different stages of DPN. Both duration of diabetes and VPT increased with increase in severity of DPN, as shown in Fig. 1H,I, respectively.

Using clinical diagnosis of DPN as a dependent variable, each clinical test index was included in an unconditional multivariate logistic regression analysis model. Potential confounding factors that may affect the geometry of the retinal vessels (such as, age, hypertension, history of smoking and drinking) were controlled for in the analysis. Duration of diabetes, VPT, fasting plasma C peptide, 24-h urinary protein excretion, 24-h urinary protein excretion rate, BCa, BCv, BAv, CRVE, DFa, and DFv showed a correlation with DPN incidence rate (α= 0.05).

After unconditional multivariate logistic regression analysis, Spearman non-parametric correlation analysis was used to assess the correlation of duration of diabetes and retinal vessel geometric parameters with DPN stage. DPN stages were positively correlated with the duration of diabetes, VPT, CRVE, and negatively correlated with DFa and DFv (Supplementary Table S2).

**Figure 2:**
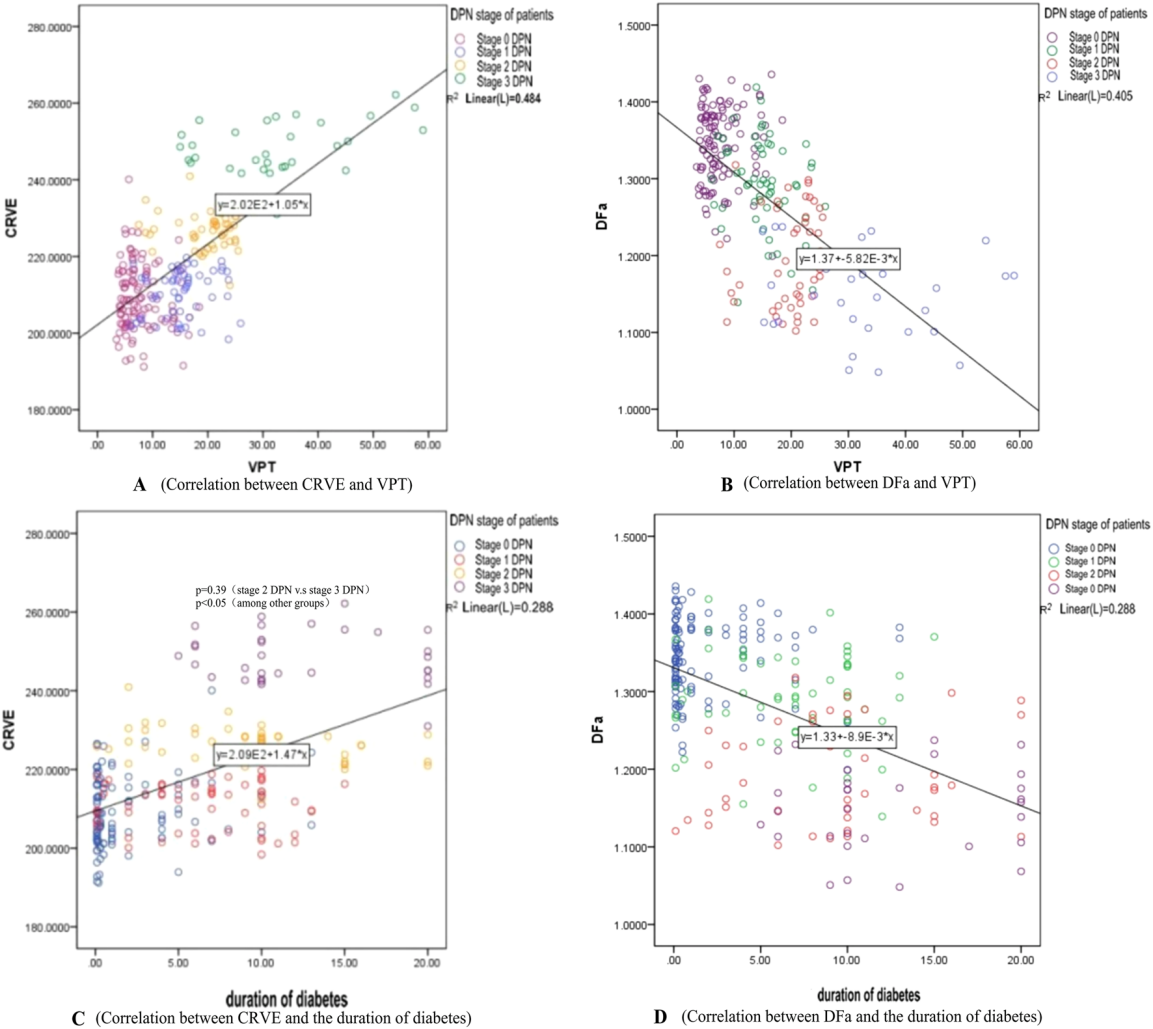
Correlation of CRVE, DFa, and VPT with duration of diabetes. *CRVE* Central retinal vein equivalent, *DFa* Fractal dimension arterioles, *VPT* Vibration perception threshold.

Using Pearson parametric correlation analysis, VPT was positively correlated with CRVE (R = 0.696) and negatively correlated with DFa (R = − 0.636), as shown in Fig. 2A,B, respectively. The duration of diabetes was positively correlated with CRVE (R = 0.534) and negatively correlated with DFa (R = − 0.537), as shown in Fig. 2C,D, respectively.

### BAa, CRAE and in interquartile of DPN incidence

Average BAa was 71.18°. The BAa Press interquartile divided into four groups: 25% percentile is 61.72°; 50% percentile is 72.19°; and 75% percentile is 77.70°, as shown in Supplementary Table S3-1. DPN incidence rate in the 4 groups were compared using χ^2^ test.

Average CRAE was 147.26 μm. The BAa Press interquartile divided into four groups: 25% percentile is 140.70 μm; 50% percentile is 147.14 μm; 75% percentile is 153.11 μm, as shown in Supplementary Table S3-2. DPN incidence rate in the 4 groups were compared using χ^2^ test.

### Retinal vascular parameters, VPT of DPN diagnostic value comparison

**Figure 3:**
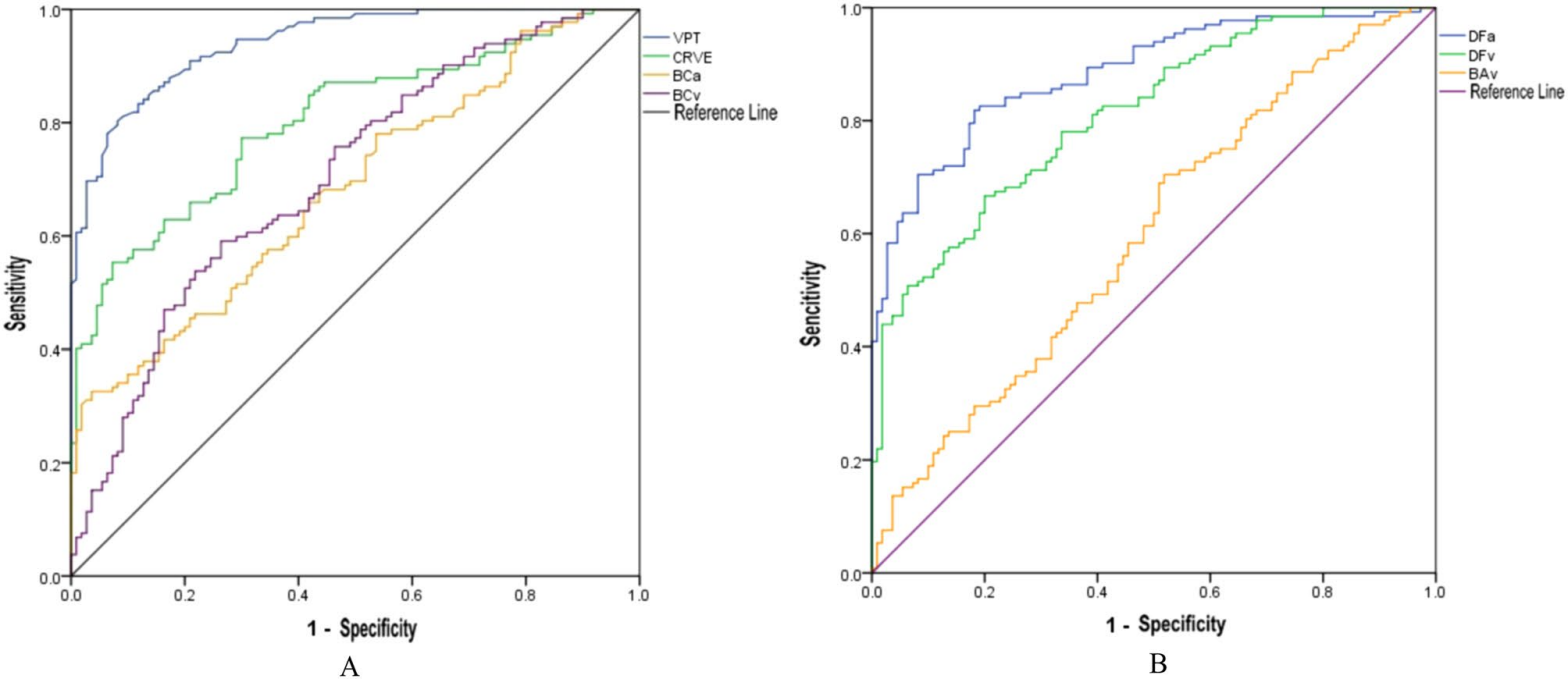
ROC curves for VPT, CRVE, BCa, BCv, DFa, DFv and BAv. *ROC* Receiver operating characteristics, *VPT* Vibration perception threshold, *CRVE* Central retinal vein equivalent, *BCa* Branching coefficient arterioles, *BAv* Branching angle veins, *BCv* Branching coefficient veins, *DFa* Fractal dimension arterioles, *DFv* Fractal dimension veins.

VPT, BCa, BCv and CRVE showed an increasing trend with increase in severity of DPN lesions. A large threshold value was used to plot the receiver operating characteristic curve (ROC) as shown in Fig. 3A.

DFa, DFv and BAv showed a decreasing trend with increase in the severity of DPN lesions. A small threshold value was used to plot the ROC as shown in Fig. 3B.

The area under the curve (AUC) of each index was used to determine the optimal diagnostic threshold value as shown in Table 2. AUC of VPT was 0.941, with the highest accuracy. AUCs of CRVE, DFa and DFV were greater than 0.8 [0.803, 0.884, 0.812 accuracy, respectively (*P* < 0.05)].

**Table 2:** AUC comparison between VPT and retinal vessel parameters.

	Area (AUC)	Standard error	*P *value	95% confidence interval
VPT	**0.941**	0.013	< 0.001	(0.915,0.968)
BCa	0.683	0.034	< 0.001	(0.617,0.749)
BCv	0.704	0.033	< 0.001	(0.639,0.769)
BAv	0.598	0.037	0.009	(0.527,0.670)
CRVE	**0.803**	0.028	< 0.001	(0.749,0.857)
DFa	**0.884**	0.021	< 0.001	(0.075,0.156)
DFv	**0.812**	0.027	< 0.001	(0.760,0.864)

Bold values indicate area under the curve greater than 0.8.

*AUC* area under the curve, *VPT* Vibration perception threshold, *BCa* Branching coefficient arterioles, *BCv* Branching coefficient veins, *BAv* Branching angle veins, *CRVE* Central retinal vein equivalent, *DFa* Fractal dimension arterioles, *DFv* Fractal dimension veins.

When using diabetic peripheral neuropathy as diagnostic criteria, ROC analysis curve was used to determine the diagnostic threshold of CRVE, DFa, DFV and VPT. The diagnostic threshold of 14.05 V for VPT was associated with 80% sensitivity and 92% specificity. The diagnostic threshold level of 220.97 μm for CRVE was associated with 55% sensitivity and 93% specificity. The diagnostic threshold of 1.30 for DFa was associated with 82% sensitivity and 82% specificity. The diagnostic threshold of 1.13 for DFv was associated with 67% sensitivity and 80% specificity (Table 3).

**Table 3:** AUC analysis of VPT and retinal vessel parameters.

	Area (AUC)	Diagnostic threshold	Sensitivity	Specificity	Youden index
VPT	0.941	14.05	0.80	0.92	0.72
CRVE	0.803	220.97	0.55	0.93	0.48
DFa	0.884	1.30	0.82	0.82	0.64
DFv	0.812	1.13	0.67	0.80	0.47

*AUC* area under the curve, *VPT* Vibration perception threshold, *CRVE* Central retinal vein equivalent, *DFa* Fractal dimension arterioles, *DFv* Fractal dimension veins.

### Diagnostic agreement between retinal vessel geometry parameters and VPT in DPN

CRVE, DFa and DFv showed good agreement with VPT for diagnosis of DPN (Kappa values: 0.470, 0.547 and 0.435, respectively; Table 4). Combined use of parameters also showed good agreement with Kappa value of 0.430.

**Table 4:** Kappa analysis of retinal vessel geometry parameters and VPT in DPN.

	Kappa values	*P *value
CRVE	0.470	< 0.001
DFa	0.547	< 0.001
DFv	0.435	< 0.001
Joint parameters	0.430	< 0.001

*VPT* Vibration perception threshold, *DPN* diabetic peripheral neuropathy, *CRVE* Central retinal vein equivalent, *DFa* Fractal dimension arterioles, *DFv* Fractal dimension veins.

Using the above-mentioned diagnostic thresholds, the rates of detection of DPN with VPT, CRVE, DFa, and DFv were 47.52%, 33.06%, 52.89% and 45.04%, respectively. The joint parameter detection rate was 64.88%. All retinal vascular geometry analysis showed significant difference in detection rate compared to VPT using Chi-squared test (Table 5).

**Table 5:** Detection rate of retinal vascular geometry compared to VPT.

	χ^2^ value	*P* value
CRVE	58.63	< 0.001
DFa	73.18	< 0.001
DFv	45.96	< 0.001
Joint parameters	50.65	< 0.001

*VPT* Vibration perception threshold, *CRVE* Central retinal vein equivalent, *DFa* Fractal dimension arterioles, *DFv* Fractal dimension veins.

### Comparison between retinal microvascular abnormalities and VPT

With CRVE > 220.97 μm, DFa < 1.30 and DFv < 1.13 as criteria for abnormal retinal microvasculature, 157 out of 242 were diagnosed as having abnormal retinal capillaries, of which 56 (35.67%) patients had normal VPT. With VPT > 14.05 V as criteria, 115 out of 242 patients were diagnosed as abnormal, of which 14 (12.17%) patients showed normal retinal capillaries (Table 6).

**Table 6:** Comparison between retinal microvascular abnormalities and VPT.

VPT	Retinal microvascular		Total
	Normal	Abnormal	
Normal	71	56	127
Abnormal	14	101	115
Total	85	157	242

*VPT* Vibration perception threshold.

## Discussion

The incidence rate of DPN was 54.5% in this study, whereas incidence rates of DPN found in previous studies have ranged between 2.4 and 74.8%^1–5^. The difference in the incidence rate may be related to demography or different diagnostic criteria. In a recent study, the prevalence rate of DPN in adults was about 53.6%^25^ excluding potential confounding factors (such as age, high blood pressure, smoking and drinking history) that may affect the geometry of the retinal vessels. Unconditional multivariate logistic regression analysis of the association between incidence of DPN and variables such as duration of diabetes, 24-h urinary protein, 24-h urinary protein excretion rate, VPT, BCa, BCv, BAv, CRVE, DFa and DFv, was performed. However, only the duration of diabetes, VPT and CRVE showed a significant positive linear correlation with the severity, whereas DFa and DFv showed a significant negative linear correlation with the severity.

The duration of diabetes has long been shown to be an independent risk factor for peripheral neuropathy. Ashok, et al.^26^ have shown that DPN was related to age and duration of diabetes. The prevalence of neuropathy in patients with DM at diagnosis was about 10%. For patients with diabetes for more than 25 years, the prevalence increased to 50%^27^. Current data showed increased population of young patients. For patients with severe DPN (Stage 2 and 3 DPN), no differences in patient age were observed, yet the duration of diabetes increased with the severity of DPN.

Bio-Thesiometer was used for quantitative evaluation of VPT reflecting functional nerve fibers and as an indicator of peripheral neuropathy. This examination is painless, simple, short, and is associated with good repeatability. Extensive clinical studies have shown the association of VPT with neuropathy^28^. We showed that VPT was correlated with the severity of DPN, in that VPT increased with the extent of nerve lesions. VPT was suggested as a diagnostic criteria for DPN in ADA guidelines 2013^14^. Yet, no explicit diagnostic criteria have been formulated. Studies have adopted different VPT thresholds as diagnostic criteria. Jayaprakash, et al*.*^29^ applied VPT ≥ 25 V as the diagnostic criterion, whereas a prospective study^30^ showed that use of VPT < 15 V found no significant DPN. In general, VPT between 16–24 V indicated DPN, and moderate risk of neuropathic ulcers; VPT > 25 V indicated serious DPN, and a high risk of neuropathic ulcers. Study also demonstrated that VPT > 25 V was an independent risk factor for foot ulcers. In this study, VPT diagnostic criteria was re-examined with ROC curve analysis.

In our study, use of VPT of 14.05 V as the diagnostic criterion was associated with 80% sensitivity and 92% specificity (AUC: 0.41). The difference in diagnostic criteria may have been responsible for the relatively lower sensitivity. In another study conducted in Indian population, lowering of VPT diagnostic threshold from 25 to 20 V increased the diagnostic sensitivity from 50 to 62.5%^31^. This may reflect the diagnostic criteria in particular enthnicities.

Diabetic microangiopathy is a specific lesion of diabetes. Retinal blood vessels represent the only vessel system amenable to direct, non-invasive assessment of vessel geometry and microvascular pathology. However, in a study^32^, type 2 diabetes patients with central retinal artery, vein and posterior ciliary artery alteration did not exhibit signs of retinopathy on fundus examination. A Danish study of 16 years of young people with type 1 diabetes (DCPD 1987)^19^ found the association of large arterioles and veins diameter with the development of neuropathy, nephropathy and proliferative retinopathy. In particular, there were a strong correlation of arteriolar narrowing and broadening of venules with microvascular disease. Thus, these changes were suggested to be early biomarkers for microvascular disease. In a study by Ding, et al.^18^, subjects with large CRAE or BCa were shown to be more likely to develop DPN. Our data elaborated further that CRAE first increases and decreases with the severity of DPN after stage 1 DPN. This might be related to the differences in pathogenesis of DPN between type 1 and type 2 diabetes. Molnar, et al.^33^ found that retinal artery stenosis occurred two to three years prior to retinal microvascular dysfunction. Retinal vascular smooth muscle cells modulate the retinal blood flow by Ca^2+^ and K^+^ channels. Early decrease of Ca^2+^ sensitivity in diabetes compared to non-diabetic subjects showed continued contraction of vascular smooth muscle cells^34^ further the loss of vasomotor dysfunction and support. Continuous vasodilation due to capillary degradation indicates the alteration of small arteries in the organs and tissues^19,35^, whereas alteration in small veins may reflect the changes in the body's compensatory mechanisms and maladaption. Expansion of retinal vein would be a good indicator of worsening of DM. Current study demonstrated that CRVE increased in late stage DPN.

Angiogenesis occurs under retinal disc hypoxia in diabetes^36^. The density of blood vessels increases and artery branch angle decreases to improve retinal perfusion^35^. Artery branch angle increases with the duration of diabetes^37^; such changes represent hemodynamic changes, endothelial dysfunction, and changes in blood oxygen carrying capacity. BAa was found to increase with DPN severity with a minor dip in early stage. This change may reflect the early development of vascular branches followed by reduction in the branches or severe vascular tortuosity upon progression of lesions. We found that BAv decreases with the increase in severity. The progression of lesions may reduce branching of the veins and increases stiffness. One the other hand, increase in branch coefficient represents increased vascular branching or vascular narrowing. Rasmussen, et al*.*^19^ found that large BCa and BCv increased the risk of diabetic nephropathy [BCa: OR 3.10, 95% CI (1.01–9.54); BCv: OR 2.11, 95% CI (1.11–4.03)], which is consistent with our findings. Fractal dimension is the degree of retinal capillary filling in the plane of retina and reflects the vascular density and complexity. Current data showed that DFa and DFv decreased with DPN severity.

In line with previous studies, we have shown that the duration of diabetes, VPT, CRVE and DFa correlated with the stage of DPN. In addition, our results showed that BAa and CRAE generally increased with increase in the severity of DPN. In the interquartile analysis, the incidence rate was smallest in the 2 middle quartiles of BAa. The incidence rate of highest quartile of CRAE was found to be double that of the lowest quartile. Of note, a few patients without DPN were included in the second quartile of BAa and CRAE. Although BAa and CRAE showed a good correlation with DPN incidence rate, sole use of these 2 parameters for DPN diagnosis might be insufficient.

In the ROC analysis, AUCs of retinal vascular geometry parameters were greater than 0.8 with high accuracy. The diagnostic threshold for each parameter was calculated for reference. *Kappa* analysis was used to assess the agreement of these diagnostic thresholds with VPT. While alteration of retinal blood vessels is a complex process associated with simultaneous changes in several parameters, CRVE, DFa and DFv were combined for DPN diagnosis. The combined use of of CRVE > 220.97 μm, DFa < 1.30 and DFV < 1.13 offered the highest DPN detection rate (64.88%), which was greater than that with use of VPT (47.52%).

Alteration of retinal vascular geometry reflects the microvasculature changes in DM patients. The pathogenesis of microvascular disease includes development of capillary wall lesions, and changes in microvascular blood flow^38^. Patients in early stages of diabetes were found to exhibit capillary damage, visible vascular endothelial cell proliferation, and intimal thickening^39^. Increased endothelial capillary permeability may result in leakage of plasma factors into the local tissue followed by changes in the nerve cell microenvironment. Intimal thickening of the capillary causes lipid deposition leading to blockage of the vessel lumen^40^. This potentially leads to local hypoperfusion and further dysfunction of nerve cells. Microvascular disease develops into neuropathy and is one of the hallmarks of diabetic peripheral neuropathy^41^. In the early stages, microvascular disease and peripheral neuropathy is largely asymptomatic; few tests are available for diagnosis. Early stages of DPN mainly involve lesions in small nerve endings and nerve fibers^42^. Symptoms include pain and loss of temperature sensation. As DPN progresses, damage to myelinated nerve fiber occurs. It is only at this stage that the abnormalities can be detected by VPT^43^. Retinal vascular parameters can be non-invasively acquired for the assessment of retinal vascular geometry, which reflect microvascular disease in other parts of the body. With CRVE > 220.97 μm, DFa < 1.30, and DFv < 1.13 as criteria of abnormal retinal microvascular, 157 out of 242 were found to have abnormal retinal capillaries; of these, 56 (35.67%) had normal VPT. With VPT > 14.05 V as criteria, 115 out of 242 were diagnosed as VPT abnormal; of these, 14 (12.17%) showed normal retinal capillaries. Overall, the assessment of retinal vascular geometry could be a good tool for early detection of microvascular abnormalities as indirect indicators of DPN.

Neuropathy scoring systems used in this study included *Michigan neuropathy screening instrument*, neurological deficit score. Yet, these allow for semi-quantitative assessment of the severity of DPN. These methods require patients to accurately describe their symptoms, and signs, which tend to be subjective in nature and dependent on the patients’ judgment. While VPT reflects the integrity of the peripheral nerves to the brain cortex in the sensory pathways, there is no spatial specificity. However retinal vascular geometric parameters could be acquired by automatic retinal fundus photography analysis system. ADA diabetes clinical guidelines 2016 recommend screening for diabetic retinopathy within 5 years of onset of type 1 diabetes in adults or at the onset of type 2 diabetes in adults High-quality fundus photography should be performed once every 2 years without retinopathy or once a year for those diagnosed with retinopathy; the frequency should be appropriately increased according to the test results. The ADA diabetes clinical guidelines 2016 also recommend VPT for screening of DPN within 5 years of the onset of type 1 diabetes in adults or at the onset of type 2 diabetes in adults. Nevertheless, current study revealed similar if not better detection rate of DPN using retinal vascular geometry analysis than VPT. Diabetic retinopathy screening by fundus photography can be used to analyze retinal vascular geometry for DPN so as to reduce the burden or medical costs on the patients. Since stage 0 DPN was not clinically diagnosed as DPN, stage 0 DPN was used as control. Current evaluation may only be limited to diabetic patients.

Diabetes duration, vibration perception threshold and retinal vascular geometry including diameter and branch angle changes were found to be related to the incidence and severity of DPN. In particular, stage of DPN showed a strong linear correlation with the duration of diabetes, VPT, CRVE, and DFa, whereas CRVE and DFa showed a linear correlation with VPT and duration of diabetes. Current study suggests retinal vascular geometry analysis for early detection of DPN. Diabetic retinopathy assessment by fundus photography may be used to analyze retinal vascular geometry for DPN.

## Supplementary Information

Supplementary Information

## Data Availability

All data generated or analyzed during this study are included in this MS.

## Abbreviations

DPN: Diabetic peripheral neuropathy
VPT: Vibration perception threshold
DFa: Fractal dimension arterioles
DFv: Fractal dimension veins
TCSS: Toronto clinical scoring system
MNSI: Michigan neuropathy screening instrument
NDS: Neurologic deficit score
DNSS: Diabetic neuropathy symptom score
ADA: American diabetes association
