# Supplementary material for: Clinical study of diabetic peripheral neuropathy screening by retinal vascular geometric parameters
Source: Sci Rep. 2021 Mar 24;11:6784. doi: 10.1038/s41598-021-85831-0 (PMC7990935; doi:10.1038/s41598-021-85831-0)
Supplement: Supplementary file 1 — Supplementary Information [file 41598_2021_85831_MOESM1_ESM.doc]

**Clinical Study of Diabetic Peripheral Neuropathy Screening by Retinal Vascular Geometric Parameters**

**Running title**: Retina Vessel Geometry for DPN Screening

Guotao Hu^1＃^, Hongmei Wu^1＃^, Lei Kuang^2^, Benny Chung-Ying Zee ^2^, Ying Huang^1^, Zhen Huang^3^, Li He^4^, Yuanhong Zeng^5^, Yongbo Gao^6^, Hailan Wang^1^*

^1^Department of Endocrinology, Longgang Central Hospital, Shenzhen 518116, Guangdong, China

^2^Department of Biostatistics, The Jockey Club School of Public Health and Primary Care, The Chinese University of Hong Kong, Shatin, New Territories, Hong Kong, China

^3^Department of Laboratory, Longgang Central Hospital, Shenzhen 518116, Guangdong, China

^4^ Department of Nursing, Longgang Central Hospital Shenzhen 518116, Guangdong, China

^5^Department of Ophthalmology, Longgang Central Hospital, Shenzhen 518116, Guangdong, China

^6^Department of Stomatology, Longgang Central Hospital, Shenzhen 518116, Guangdong, China

^＃^Contributed equally

*Corresponding author: Hailan Wang

Department of Endocrinology, Longgang Central Hospital, Shenzhen 1228 Longgang Road, Shenzhen, 518116,Guangdong,China

Email: [13670298213@163.com](mailto:13670298213@163.com)

Tel: +86-13670298213

Supplementary Table 1 Measurement method of VPT and retinal vessel geometric parameters

| Parameter | Method | Tools |
| --- | --- | --- |
| Vibration perception threshold (VPT) | Patients were allowed to familiarize themselves with the vibratory sensation and to inform the examiner as soon as they perceived the vibratory sensation without much adaptation. The rubber tactor was balanced vertically on the pulp of the big toe. The voltage was increased from 0V until the patient perceived the vibratory sensation. The test was repeated 3 times on each foot. The average VPT of both feet was recorded. | Bio-Thesiometer quantitative sensiometer |
| Central retinal artery equivalent (CRAE) | The diameter of 6 broadest arterioles found in the range of 0.5–2 optic disc diameter. CRAE reflects changes in the caliber of retinal arteries. | Singapore "I" Vessel Assessment, SIVA |
| Central retinal vein equivalent (CRVE) | The diameter of 6 broadest veins found in the range of 0.5–2 optic disc diameter. CRVE reflects changes in the caliber of retinal veins. |
| Branching Angle  arterioles (BAa) | The angle of the upper two sub arteries bifurcation. Branch angle (normal value: approximately 75°) is associated with the number of vascular branches and the degree of tortuousity. Changes in BAa suggest hemodynamic changes, endothelial dysfunction, or altered blood oxygen concentration. |
| Branching Coefficient arterioles (BCa) | The ratio between the square of diameter of the central artery and the square diameter of the superior artery. Increased branching coefficient represents increased branch blood vessels or superior vascularization. |
| Branching Angle  veins (BAv) | The angle of the upper two sub veins bifurcation. |
| Branching Coefficient veins (BCv) | The ratio between the square of diameter of the central vein and the square diameter of the superior vein. |
| Fractal Dimension arterioles (DFa) | The degree of filling of the retinal arterioles in the retinal plane. DF reflects the complexity of vascular and vessel density; the greater the value the more complex the structure. |  |
| Fractal Dimension veins (DFv) | The degree of filling of the retinal veins in the retinal plane. |

Supplementary Table 2 Correlation of duration of diabetes, VPT, and retinal vessel geometric parameters with DPN stage

|  | Correlation coefficient (R) | *P* value |
| --- | --- | --- |
| Duration of diabetes | **0.706** | <0.001 |
| VPT | **0.818** | <0.001 |
| CRVE | **0.716** | <0.001 |
| DFa | **-0.769** | <0.001 |
| DFv | -0.617 | <0.001 |

VPT, Vibration perception threshold; CRVE, Central retinal vein equivalent; DFa, Fractal dimension arterioles; DFv, Fractal dimension veins

Supplementary table 3-1 BAa and inter-quartile of DPN stages

| BAa quartile | N | DPN incidence | Stage 1 | Stage 2 | Stage 3 | *χ*^2^value | *P*value |
| --- | --- | --- | --- | --- | --- | --- | --- |
| 41.77–61.72 | 60 | 68.33% | 68.29% | 21.95% | 9.76% | 20.86 | <0.001 |
| 61.72–72.19 | 61 | 37.70% | 52.17% | 39.13% | 8.70% |
| 72.19–77.70 | 61 | 42.62% | 30.77% | 38.46% | 30.77% |
| 77.70–106.10 | 60 | 70.00% | 19.05% | 40.47% | 40.48% |

BAa, Branching angle arterioles; DPN, diabetic peripheral neuropathy

Supplementary table 3-2 CRAE and inter-quartile of DPN stages

| CRAE quartile | N | DPN incidence | Stage 1 | Stage 2 | Stage 3 | *χ*^2^value | *P*value |
| --- | --- | --- | --- | --- | --- | --- | --- |
| 131.07–140.70 | 60 | 31.67% | 86.96% | 13.04% | 0 | 16.55 | 0.001 |
| 140.70–147.14 | 61 | 62.30% | 68.42% | 28.95% | 2.63% |
| 147.14–153.11 | 61 | 52.46% | 28.12% | 62.50% | 9.38% |
| 153.11–166.86 | 60 | 65.00% | 2.56% | 28.21% | 69.23% |

CRAE, Central retinal artery equivalent; DPN, diabetic peripheral neuropathy
